# The Pathogenesis of Disinhibition in Patients with Traumatic Brain Injury: A Two Patient Case Report

**DOI:** 10.3390/brainsci13081227

**Published:** 2023-08-21

**Authors:** Takashi Hiraoka, Masami Yagi

**Affiliations:** 1 Department of Rehabilitation Medicine, Kawasaki Medical School, Kurashiki City 701-0192, Japan; 2 Rehabilitation Center, Kawasaki Medical School Hospital, Kurashiki City 701-0192, Japan; am-yagi@med.kawasaki-m.ac.jp

**Keywords:** higher brain dysfunction, traumatic brain injury, social behavioral impairment, disinhibition, impulse control disorder, unlawful act, suicide

## Abstract

Higher brain dysfunction commonly occurs following traumatic brain injury (TBI), and may manifest in a social behavioral impairment which can significantly impede active social participation. We report two cases, one of voyeurism and the second of alcohol abuse, which might have been caused by TBI resulting in disinhibition, a type of social behavioral impairment. We discuss the underlying pathophysiological mechanisms to raise awareness of such cases and aid the development of effective interventions. Patient 1 suffered a TBI at 18 years of age, 2 years after which he presented repeated episodes of sexually deviant behavior (voyeurism). At 28, he committed suicide, since he was unable to control his aberrant behavior. Patient 2 suffered a TBI at the age of 13. He first displayed problematic behavior 7 years later, which included drinking excessive amounts of alcohol and stealing while inebriated. Despite both patients having sound moral judgment, they had irrational and uncontrollable impulses of desire. Imaging findings could explain the possible causes of impulse control impairments. Damage to the basal ganglia and limbic system, which are involved in social behavior, presumably led to desire-dominated behavior, leading to the patients conducting unlawful acts despite intact moral judgment. It is crucial to educate society about the prevalence of these disorders, explain how these disinhibitions start, and develop effective interventions.

## 1. Background

“Higher brain dysfunction” is a diagnostic and administrative term unique to Japan [1]. It has clear diagnostic criteria and can be described as “a condition involving difficulty in adapting to daily and social life mainly due to a cognitive disorder such as memory disorder, attention disorder, executive dysfunction, and social behavioral impairment due to organic lesions with a confirmed cause, including traumatic brain injury (TBI) or cerebrovascular disease”. The concept is similar to “organic mental disorder” in psychiatry, which includes the ICD (International Statistical Classification of Diseases and Related Health Problems) codes F04, F06, or F07. Social behavioral impairment, which is a type of higher brain dysfunction, can significantly limit a patient’s active participation in society. Symptoms of social behavior impairment include dependency/regression, low impulse control, decreased emotional control, poor interpersonal skills, persistence, decreased motivation/spontaneity, depression, emotional incontinence, withdrawal, disinhibition, paranoia, and wandering [2]. Specifically, disinhibition (the inability to control one’s actions and emotions), including decreased impulse or emotional control, can impair one’s ability to perform social roles. Although there have been previous reports, including the most recent [3], that have discussed the relationship between traumatic brain injury and social behavioral disorders, most have been concerned with the relationship between traumatic brain injury and emotional dysregulation such as irritability, anger, etc. Some studies [4,5,6,7,8] have addressed the possibility that “traumatic brain injury” can cause “illegal acts associated with reduced impulse control” as described in the present research article, and almost no papers can be found that detail the mechanisms involved. As noted above, decreased impulse control can lead to the commission of various unlawful acts, including molestation and theft. Criminal insanity due to higher brain dysfunction has been recognized as a nationwide problem and discussed by the National Liaison Council for Higher Brain Dysfunction Support. Further, “Response and Support for People with Social Behavior Disorders” was compiled as a topic in the 2016–2018 Ministry of Health, Labour, and Welfare scientific research on the response to difficulties in social participation due to social behavior disorders among people with higher brain dysfunction [9]. According to this report, overdrinking and overeating (33%), shoplifting (13%), suicide and attempted suicide (15%), and sexual deviation (9%) were prevalent symptoms among individuals with higher brain dysfunction. Various other papers have reported on the relationship between social cognition and social behavior after traumatic brain injury, including reports describing the causes of social behavior disorders [10]. However, there are few detailed case reports of patients with higher brain dysfunction who have behavioral problems; moreover, the mechanisms underlying the pathogenesis remain unclear [6]. Generally, behavioral characteristics, such as irritability or anger, are easily perceived by those around the patients and have a significant impact on the patient’s social life. However, it might be challenging for society to recognize that some people have issues with their social lives, such as irrepressible illegal acts, despite not having emotional intolerance (irritability or anger). It is crucial to educate society about these disorders, and we feel that it is our duty as specialists in higher brain dysfunction to do so. Accordingly, this article describes two cases of criminal insanity in patients with higher brain dysfunction and discusses the potential underlying mechanisms, which could help guide future research. Informed consent to publish the manuscript was obtained from both the patient and his parents in Case 2 and only the parents in Case 1 (due to the death of the patient).

## 2. Case Presentation

### 2.1. Case 1

**Figure 1:**
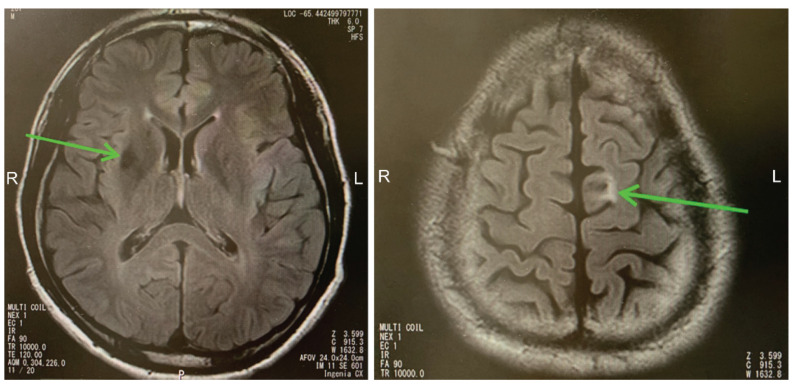
Brain magnetic resonance imaging (MRI) scans of Patient 1 show bilateral diffuse axonal injury (DAI) of the frontal and temporal lobes, as well as DAI of the right basal ganglia, splenium of the corpus callosum, and midbrain. Green arrows indicate major brain injury lesions.

Patient 1 was a man who committed suicide after experiencing repeated episodes of sexual deviance. The patient was born healthy and had no remarkable medical history. He had acquired 12 years of education and had not exhibited any aberrant behavior, including sexually deviant behavior, prior to experiencing a TBI. At the age of 18 years, he was involved in a traffic accident and suffered a diffuse axonal injury (DAI); occipital bone fracture; acute epidural hematoma of the posterior fossa; multiple fractures of the mandible, upper and lower limbs, and spine; and disturbance of consciousness for approximately 1 month. Upon arrival at the emergency department, he had a Glasgow Coma Scale (GCS) score of E1V1M4; further, he immediately underwent endotracheal intubation due to tongue-base depression. He underwent a tracheostomy on day 14 of hospitalization and was transferred to our hospital for rehabilitation on day 43. On the 44th hospitalization day, he could follow simple verbal commands. Further, he was switched to a speech cannula and could speak briefly and weakly. No complicated behaviors were observed during his hospital stay. He received inpatient treatment for approximately 6 months in the acute care hospital and subsequently in the Convalescent Rehabilitation Ward of our hospital, and was discharged on the 221st day upon regaining his ability to independently perform his activities of daily living (ADL). Multiple neuropsychological examinations (Table 1) revealed that despite persistent mild attention disorder, the patient recovered normal intellectual function, with no further deterioration. His cognitive function was at the lower limit of normal, except for processing speed, on the Wechsler Adult Intelligence Scale 3rd edition (WAIS-III). The memory and executive function tests were within normal limits. Brain magnetic resonance imaging (MRI) performed 1 year after the injury (Figure 1) revealed a bilateral DAI of the frontal and temporal lobes, as well as a DAI of the right basal ganglia, splenium of the corpus callosum, and midbrain.

After discharge, he visited our outpatient clinic for higher brain dysfunction management and for help finding employment. The type of higher brain dysfunction that the patient was diagnosed with was mainly attention disorder due to observed residual episodes of the disorder while at home. He was mellow and friendly; further, he could appropriately answer the physician’s questions. This suggested that he could return to work without any problems, with some accommodation from the employer for mild attention disorder and mild left leg paresis. Accordingly, the patient returned to work approximately 14 months after the injury and showed good performance. However, approximately 26 months after the injury (12 months after returning to work), he was suspended for secretly video-recording inside the women’s restroom at his workplace. During the suspension, he had difficulty controlling his appetite and gained weight. Although he was allowed to return to work, he had more episodes of camera voyeurism inside the women’s locker room and was dismissed approximately 3 years after returning to work. Subsequently, after obtaining employment in various workplaces, he demonstrated a positive attitude toward social participation, including joining a blind soccer team as a hobby, which gave a superficial impression of healthy interpersonal functions. However, he still had repeated incidents of peeping, voyeurism, and molestation (touching a colleague’s buttocks) during his 1–2 years at work or when commuting to work. Despite a high level of intellectual ability, general interpersonal skills, and social adaptability, his sexually deviant behavior prevented him from obtaining employment. After each incident, he would express sincere remorse during medical examination, stating that peeping, voyeurism, and molestation are criminal acts that are unacceptable in society, and would say, “I am sorry for my behavior towards the victims”, assuring never to repeat the act. However, he would simultaneously appear to rationalize his actions with statements such as “I thought that the victims were asking for it; I thought that they wanted to be touched”. Accordingly, he continued to repeatedly commit acts of sexual deviation. He described his psychological state as follows: “I am not bothered by my inability to suppress my sexual desires; instead, I am bothered by the subsequent punishment, such as suspension”. Notably, he tried to obtain the phone numbers of women hospital staff immediately after expressing his remorse, indicating the severity of his compulsive sexual behavior. Accordingly, it was considered essential to place restrictions on his social participation by prohibiting the possession of devices with cameras, being driven to and from work by his parents, and staff at work monitoring his behavior. Nonetheless, the patient was arrested by the police several times and was fired by his company. Moreover, his engagement ended and he was scorned by his colleagues and friends.

Approximately 8 years after the TBI, his parents allowed him to acquire a camera-equipped smartphone for work purposes. However, he was soon arrested for secretly filming a woman while bathing. Subsequently, he began to fall into depression, stating that “I want to die so that I do not cause any more trouble for my family”. The patient was presented to a psychiatric hospital but was not hospitalized. While recovering at home, he escaped and committed suicide by drowning at a port near his home. Later, analyzing his computer, his parents found a large number of previously unseen voyeuristic images and a history of purchasing voyeuristic tools online.

### 2.2. Case 2

Patient 2 is a man currently on criminal trial for repeated theft associated with alcohol abuse. The patient was born healthy and had no remarkable medical history. He had been educated for 12 years and suffered a TBI in a traffic accident at the age of 13. Upon arrival at our University Hospital, the patient was conscious (GCS score E1V1M3), and he was immediately orally intubated and administered hypothermia. A tracheostomy was performed on day 5 of hospitalization. On the 7th hospitalization day, his GCS score improved to E3VtM6 and he could follow simple instructions. On day 10 of hospitalization, the tracheostomy tube was removed and the patient was discharged from the intensive care unit with a GCS score of E4V4M5. On day 15, the patient was transferred to the Department of Rehabilitation Medicine. He was discharged after 52 days of hospitalization with ADL independence and slightly impaired memory.

The patient subsequently returned to middle school and began working after graduating from high school. However, his middle- and high-school grades were poor. Further, he was fired from work soon after joining due to poor performance and went on to take up temporary odd jobs. He began to consume alcohol regularly after the age of 20 as a social enhancer and a means of reducing work stress. However, he would drink in excess and started to exhibit aberrant behavior; consequently, his parents took him to our Higher Brain Dysfunction Outpatient Clinic at the age of 30 years (approximately 17 years after the TBI) for the first time. According to his parents, he had a history of troublesome episodes involving refusals to pay after binge drinking and had once stolen a truck and driven it home. However, when he was sober, he did not have such episodes. He was a dedicated worker in a facility for the elderly (a welfare facility that employs people with disabilities) and had a good reputation among his colleagues at work.

**Figure 2:**
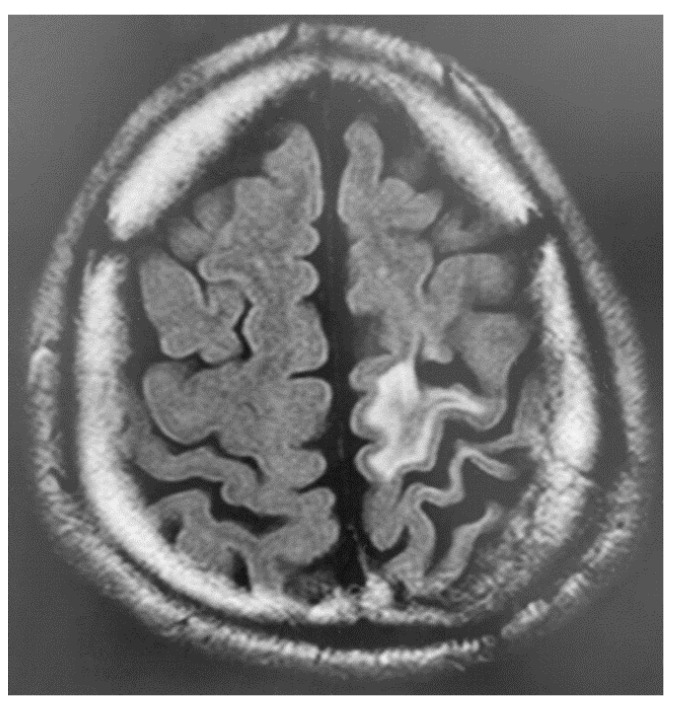
Brain MRI scan of Patient 2 (fluid-attenuated inversion recovery) shows high signal intensity and atrophy in the left frontal and parietal lobes, suggesting old brain injury.

**Figure 3:**
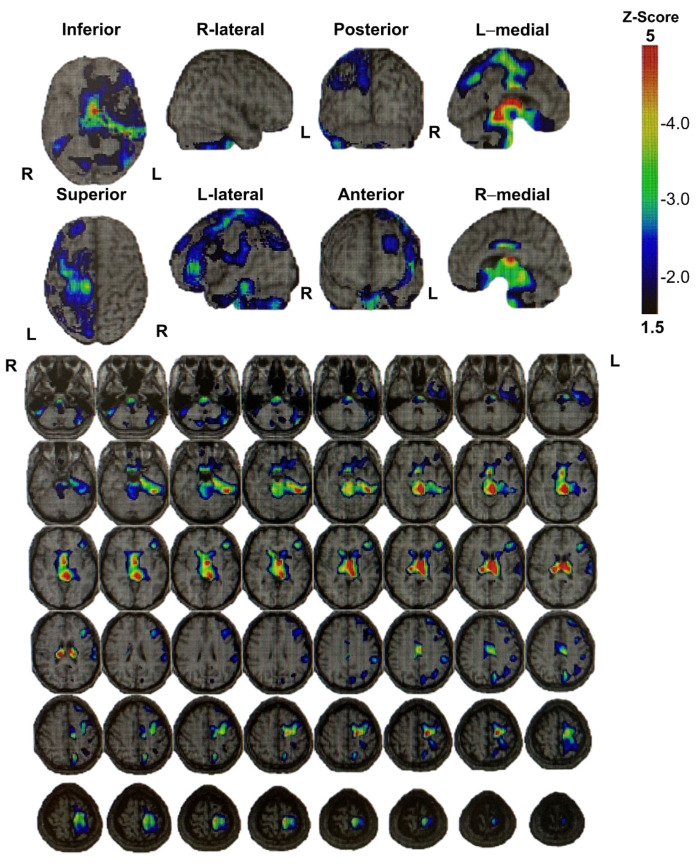
I-123-iodoamphetamine single-photon emission computed tomography images of Patient 2 show decreased blood flow in the region from the left frontal lobe to the parietal lobe, as well as bilaterally decreased blood flow in the basal ganglia (near the thalamus) and brain stem (near the midbrain).

During the medical examination, the patient expressed sincere remorse over the alcohol-related problems and desired to seek solutions. Accordingly, we started a detailed examination for higher brain dysfunction and requested a psychiatric assessment for specialized treatment of alcohol dependence/abuse. The patient was diagnosed with higher brain dysfunction based on social behavioral deficits in daily life and memory deficits confirmed by neuropsychological testing. Figure 2 and Figure 3 show the results of brain imaging performed on his first visit to our department. MRI revealed cerebral atrophy in the region from the left frontal lobe to the parietal lobe; furthermore, SPECT (Single Photon Emission Computed Tomography) confirmed decreased blood flow in the region from the left frontal lobe to the parietal lobe, as well as bilaterally decreased blood flow in the basal ganglia (near the thalamus) and brain stem (near the midbrain). Table 2 shows the results and timeline of the neuropsychological tests performed. His intelligence was consistently at the lower limit of normal from the examination 1 year after the injury to the most recent examination 23 years after injury, and his memory impairment was consistently conspicuous at all examination time points. Other tests showed no apparent decline. He consumed alcohol while receiving treatment for alcoholism in an alcoholic rehabilitation facility, after stealing alcoholic drinks from a convenience store. Currently, his alcohol impulse control disorder persists with repeated episodes of alcohol consumption and difficult behavior after sneaking away from home. At the age of 38 years, he was caught shoplifting at a home improvement store while sober and is currently on trial for impulsive behavior regardless of alcohol consumption. He has been arrested many times, with his mother expressing her frustration saying, “It would have been better if he died in the car accident if he was going to cause so much trouble to others”.

## 3. Instruments Used for Brain Imaging

The MRI scanner used was a 3.0 T MRI scanner (Vantage Titan 3 T; Canon Medical Systems, Otawara City, Japan) with a 16-channel phased array coil for case 1 and a 3.0 T MRI scanner (Ingenia 3.0 T CX Quasar Dual; Philips Healthcare, Best, The Netherlands) with a 20-channel phased array coil. An E-CAM (Canon Medical Systems, Otawara City, Japan) was used for SPECT in case 2, and easy Z-score imaging system (eZIS) (PDRadiopharma Inc, Tokyo, Japan.) was used as statistical analysis software.

## 4. Discussion and Conclusions

As mentioned earlier, both cases showed irresistible impulse suppression disorders. The existence of such cases, which may be the result of a past TBI, remains largely unfamiliar to society. Both patients were able to determine whether their activities were right or erroneous, but despite their wish to stop repeating their problematic behaviors, no actual improvements were observed. This indicated that they could not resist temptation and were unable to restrain their irrational desires. This problematic behavior can be presumably attributed to TBI-induced impulse control disorder (disinhibition). Additionally, they had a shortsighted mindset; for instance, they did not acknowledge that gratifying their immediate physiological needs would lead to severe consequences, including legal punishments. Possible mechanisms underlying the onset of these behavioral problems are discussed below.

### Mechanism Underlying the Pathogenesis

The onset of disinhibition could be attributed to injury of the basal ganglia, which was observed on MRI/SPECT in both cases. Components of the basal ganglia include the corpus striatum (the putamen, caudate nucleus, and nucleus accumbens (located at the inferior end of the corpus striatum)), globus pallidus/subthalamic nucleus/substantia nigra, red nucleus, and lateral vestibular nucleus [11]. Both cases may have suffered damage to the nucleus accumbens, which is the pleasure center. Damage to the nucleus accumbens, orbitofrontal cortex (OFC), or inferior prefrontal cortex circuit (behavioral inhibition system), as part of the frontal basal ganglia thalamic circuit [12], may have resulted in the observed sexual disinhibition and alcohol abuse (dependence/addiction). There has been a growing interest in the indirect and hyper-direct pathways of the cerebral cortex–basal ganglia circuit as crucial neurological basis for behavioral inhibition in humans [13,14,15,16], which could have resulted in poor behavioral control in both cases. Alternatively, impulse control disorders could be linked to the nigrostriatal dopaminergic system. Under normal conditions of impulse control by the nigrostriatal dopamine system, the activity of dopamine neurons in the compact part of the substantia nigra increases in response to inappropriate behavior. This leads to an increase in neuronal activity in the caudate nucleus, a striate area that receives projections from the aforementioned dopamine neurons, which inhibits inappropriate behavior. Conversely, dysfunction of the dopamine system in the pars compacta of the substantia nigra or caudate nucleus results in an inability to suppress inappropriate behavior through these pathways [17]. In Case 1, MRI revealed DAI in the midbrain and right caudate nucleus; moreover, SPECT in Case 2 showed decreased blood flow around the midbrain. Therefore, the inability to suppress inappropriate actions could be attributed to a failure of the aforementioned system.

Moreover, the A10 neuron group in the midbrain partially projects to a part of the limbic system. These neuronal groups are known as the “midbrain dopaminergic system”, and have psychological effects in response to pharmacological agents [11,18]. The A10 neuron group is located ventral to the interpeduncular nucleus in the ventral tegmental area. Substances such as alcohol, opioids, and cocaine activate this reward system to stimulate specific regions of the mesolimbic system, which yields a rewarding sensation. Therefore, this mechanism is considered the neural basis of drug dependence [18]. Although we could not elucidate how the failure of the A10 neuron system affected both cases, it may have contributed to the observed sexual deviation and alcohol abuse.

Additionally, it is important to consider the influence of the limbic cortex, even though neither patient showed obvious damage to this region. The limbic cortex is mainly involved in instincts such as appetite, sex drive, desire to sleep, and motivation, as well as emotional expression, memory, and autonomic nervous activity. Currently, there is no established definition of the neural structures that comprise the limbic cortex [19,20]. In this article, we will consider the limbic cortex as a collection of highly interconnected regions located in the inner brain, including all cortical regions along the medial edge of the cortical mantle [21], adjacent cortex, and subcortical structures [22,23,24,25,26]. These regions include the OFC, insular cortex, anterior and posterior cingulate cortex, temporal cortex, parahippocampal gyrus, hippocampus, amygdala, basal forebrain, anterior thalamic nucleus, and hypothalamus. In patients with TBI, DAI without imaging abnormalities frequently results in limbic–cortical dysfunction. This commonly occurs after traffic accidents involving disturbance of consciousness and may not necessarily be accompanied by abnormal imaging findings [27]. Pathological findings of DAI are often observed in focal brain injuries, including acute subdural, acute epidural, and intracerebral hematoma. Disturbance of consciousness immediately after injury should be considered suggestive of concurrent DAI [28]. Since both patients exhibited disturbance of consciousness in the acute period, there could have been DAI in the deep brain. Specifically, limbic cortical dysfunction in the deep cerebral region, including the OFC communication fibers, could have occurred despite the lack of abnormalities in imaging. Damage to these areas may result in behavioral patterns resembling the disinhibited behavior of frontal lobe syndrome.

In addition, the pathology in both cases could have been partly attributed to a decline in intellectual capacity; indeed, both patients showed borderline intellectual functioning (executive functioning disorder). Higher brain dysfunction, particularly frontal lobe dysfunction, can be broadly classified into executive functioning disorder and mental health dysfunction, including disinhibition [29].

Intellectual function disorder following TBI has been shown to improve over time, which was observed in both of the presented cases. However, the improvement of intellectual function does not solely guarantee the fulfillment of social roles. Impulse control disorder, which was the largest obstacle to social participation for both patients, is classified as a motivational salience dysfunction [29]. Unlike executive functioning disorder, motivational salience dysfunction typically appears after discharge from the hospital during resumption of social participation. Since social behavioral impairment manifests itself in the community, it should be addressed when it enters the chronic stage [30]. Further, social behavior disorders can greatly affect an individual’s quality of life in society [31], and thus they significantly affected the daily lives of both patients. In both cases, the impulse control disorders appeared to have manifested in an environment-dependent manner. There is a need to increase awareness regarding social behavioral impairment, including impulse control disorder, and its manifestations in the community at large.

Obviously, there is no evidence that these two cases are the result of such a mechanism, but our findings imply that favorable results in quantitative neuropsychological tests do not always portend a favorable clinical outcome or mild brain injury. Instead, social behavioral disorder may occur as a sequelae of TBI, including DAI, hindering social participation. Our patients experienced extensive agony as a result of their inability to control their instinctive urges despite knowing that their behaviors were wrong, with Patient 1 eventually committing suicide.

Moreover, our findings indicate that recidivism cannot be prevented in insane individuals with higher brain dysfunction by just punishing them under the penal code of the country. Additionally, it is also not possible to openly accept unlawful acts by criminally insane individuals. Accordingly, this remains an important challenge that needs to be addressed through society-level discussions. Moreover, it is important to elucidate the molecular mechanisms underlying these disorders for development of future treatment interventions, including drug therapy, rehabilitation medicine, and regenerative medicine.

## Figures and Tables

**Table 1:** Changes in the neuropsychological test results of Patient 1.

		Day of Injury + 6 M	Day of Injury + 1 Y	Day of Injury + 7 Y
**WAIS-Ⅲ**	VIQ	92	92	-
	PIQ	74	79	-
	FIQ	82	85	-
	VC	86	90	-
	PO	79	87	-
	WM	105	94	-
	PS	54	54	-
**WMS-R**	Verbal memory	-	118	99
	Visual memory	-	105	109
	General memory	-	117	101
	Attention	-	114	111
	Delayed recall	-	115	99
**BADS**		-	Above average	-
**FAB**		17/18	-	18/18
**TMT-A**		88 s	-	62 s
**TMT-B**		145 s	-	66 s

Abbreviations: M, month; Y, year/years; WAIS-III, Wechsler Adult Intelligence Scale 3rd edition; VIQ, Verbal Intelligence Quotient; PIQ, Performance Intelligence Quotient; FIQ, Full-Scale Intelligence Quotient; VC, Verbal Comprehension; PO, Perceptual Organization; WM, Working Memory; PS, Processing Speed; WMS-R, Wechsler Memory Scale-Revised; BADS, Behavioral Assessment of the Dysexecutive Syndrome; FAB, Frontal Assessment Battery; TMT, Trail Making Test; s, seconds.

**Table 2:** Demographic and neuropsychological assessment.

		Day of Injury + 1 Y	Day of Injury + 17 Y	Day of Injury + 23 Y
**WISC-R**	VIQ	80	-	-
	PIQ	77	-	-
	IQ	77	-	-
**WAIS-Ⅲ**	VIQ	-	82	84
	PIQ	-	70	84
	FIQ	-	74	83
	VC	-	84	90
	PO	-	72	89
	WM	-	81	83
	PS	-	66	75
**WMS-R**	Verbal memory	-	66	69
	Visual memory	-	67	71
	General memory	-	61	65
	Attention	-	76	86
	Delayed recall	-	50	50
**IGT**	-	-	-	¥147,500 (insufficient understanding of rules)
**FAB**	-	-	-	16/18
**TMT-A**	-	-	-	42 s
**TMT-B**	-	-	-	96 s

Abbreviations: Y, year/years; WISC-R, Wechsler Intelligence Scale for Children-Revised; WAIS-Ⅲ, Wechsler Adult Intelligence Scale 3rd edition; IQ, Intelligence Quotient; VIQ, Verbal Intelligence Quotient; PIQ, Performance Intelligence Quotient; FIQ, Full-Scale Intelligence Quotient; VC, Verbal Comprehension; PO, Perceptual Organization; WM, Working Memory; PS, Processing Speed; WMS-R, Wechsler Memory Scale-Revised; IGT, Iowa Gambling Task; FAB, Frontal Assessment Battery; TMT, Trail Making Test; s, seconds.

## Data Availability

Not applicable.
